# Comparing the diagnostic performance of radiation dose-equivalent radiography, multi-detector computed tomography and cone beam computed tomography for finger fractures – A phantom study

**DOI:** 10.1371/journal.pone.0213339

**Published:** 2019-03-05

**Authors:** Carolin Sophie Reidelbach, Sebastian Moritz Goerke, Simon Carl Leschka, Claudia Neubauer, Martin Soschynski, Florian Lampert, Horst Zajonc, Elmar Kotter, Mathias Langer, Jakob Neubauer

**Affiliations:** 1 Department of Radiology, Medical Center—University of Freiburg, Faculty of Medicine, University of Freiburg, Freiburg, Germany; 2 Department of Radiology, Ortenau Klinikum Offenburg-Gengenbach, Offenburg, Germany; 3 Department of Radiology, RKK Klinikum—St. Josefskrankenhaus, Freiburg, Germany; 4 Department of Plastic and Hand Surgery, Medical Center—University of Freiburg, Faculty of Medicine, University of Freiburg, Freiburg, Germany; Johns Hopkins School of Medicine, UNITED STATES

## Abstract

**Purpose:**

To compare the diagnostic performance and raters´confidence of radiography, radiography equivalent dose multi-detector computed tomography (RED-MDCT) and radiography equivalent dose cone beam computed tomography (RED-CBCT) for finger fractures.

**Methods:**

Fractures were inflicted artificially and randomly to 10 cadaveric hands of body donors. Radiography as well as RED-MDCT and RED-CBCT imaging were performed at dose settings equivalent to radiography. Images were de-identified and analyzed by three radiologists regarding finger fractures, joint involvement and confidence with their findings. Reference standard was consensus reading by two radiologists of the fracturing protocol and high-dose multi-detector computed tomography (MDCT) images. Sensitivity and specificity were calculated and compared with Cochrane´s Q and post hoc analysis. Rater´s confidence was calculated with Friedman Test and post hoc Nemenyi Test.

**Results:**

Rater´s confidence, inter-rater correlation, specificity for fractures and joint involvement were higher in RED-MDCT and RED-CBCT compared to radiography. No differences between the modalities were found regarding sensitivity.

**Conclusion:**

In this phantom study, radiography equivalent dose computed tomography (RED-CT) demonstrates a partly higher diagnostic accuracy than radiography. Implementing RED-CT in the diagnostic work-up of finger fractures could improve diagnostics, support correct classification and adequate treatment. Clinical studies should be performed to confirm these preliminary results.

## Introduction

When it comes to trauma of the hand, finger fractures belong to the most common fractures [1]. If a fracture is suspected, radiography is carried out first [2]. CT imaging, however, has been shown to be more accurate than radiography regarding diagnosis of certain fractures, fracture dislocation and joint involvement [3–5]. The main disadvantage of these techniques is their high radiation dose [6]. Lately, technical improvements such as iterative reconstruction or tube current modulation allowed for significant reduction of radiation dose in CT examinations, consequently enabling low-dose examinations in different regions of the body [7–11]. Several studies showed that low-dose imaging of the wrist is feasible with multi-detector computed tomography (MDCT) [12] and cone beam computed tomography (CBCT) [13,14]. CBCT was initially established in maxillofacial imaging and differs from the MDCT mainly through its detector [15]. The main difference between the detectors is that the CBCT's flat-panel detector has no anti-scatter-grid, which makes it more prone to artifacts from scatter radiation. Also, the pixel size of the flat-panel detector is smaller, resulting on the one hand in a higher spatial resolution, but on the other hand causing more noise [16]. CBCT is an imaging modality currently on the rise in musculoskeletal radiology [14,17–19].

Considering all the technical improvements achieved in the last few years, the purpose of this study was to lower the dose of MDCT and CBCT examinations to that of radiography, thus improving diagnostics whilst maintaining radiation doses. If it was possible to approximate the dose of MDCT and CBCT to that of radiography whilst achieving an adequate diagnostic accuracy and image quality, the radiography equivalent dose computed tomography (RED-CT) methods could be used as an alternative to radiography in clinical settings. In a previous study, RED-CT has already been shown to have a similar and in some parts even better diagnostic quality for carpal fractures than radiography [20].

The hypothesis of this study is that radiography equivalent dose multi-detector computed tomography (RED-MDCT) and radiography equivalent dose cone beam computed tomography (RED-CBCT) also have a higher diagnostic accuracy and lead to higher reader certainties compared to radiography in the diagnostics of finger fractures. Hence, we compared the performance of radiography to RED-MDCT and RED-CBCT for finger fractures, joint involvement and rater´s confidence in a multi-reader study on hand cadavers.

## Materials and methods

The Ethics Commission of the University of Freiburg approved this prospective study.

### Cadaveric hands

As study objects we used 10 cadaveric hands from volunteer body donors of our university´s Department of Anatomy. The cadaveric specimens were fixed in formaldehyde. Fractures were assigned by two hand surgeons (20 and 4 years of experience) in an operating theatre under fluoroscopic control. The decision whether to fracture a bone or not was made by rolling the dice for each finger bone (1 = fracture, 2–6 = no fracture). This process was performed from digitus 1 to 5, each starting with the proximal bone. The exact location of the fracture in the individual bone was determined by the hand surgeon inflicting the fractures. Fractures were placed using blunt orthopedic instruments. An assistant documented the procedures in a log (fracturing protocol). The cadaveric specimens were sprinkled with sodium chloride liquid during the fracturing process and all skin injuries were closed by sutures to reduce the likelihood of emphysema. Radiography as well as RED-MDCT and RED-CBCT were performed immediately afterwards. The stiffness of the cadaveric hands made it impossible to bend single fingers and keep them in a bent position. Therefore, p.a. (posterior anterior) and oblique x-rays of the entire hand were performed instead of x-rays of each finger in two planes. Reference standard was a combination of a consensus reading of high-dose MDCT and the fracturing protocol.

### Determination of dose and imaging protocols

Prior to this study, dose calculations were performed for all devices [21]. We used a radiation dose calculation system (GMctdospp) based on Monte Carlo simulations. The different examination methods were integrated into the simulation. In the experiment and the simulations a test phantom was used for calibration and measurements. The voxel phantom of a human hand, which is segmented into individual organ regions (bone, muscle, fat …), was used to simulate radiation exposure to the hand. The sum of all organ doses was used for the dose comparison. First, the radiation exposure of the radiography examination at standard settings was determined. Then the MDCT and CBCT examination protocols were adapted to apply an equivalent dose of radiation. We performed the imaging in equivalent dose settings based on these calculations.

RED-MDCT (AquilionOne, Toshiba, Otawara-shi, Japan) was performed in a 180-degree rotation volume mode (single shot without pitch). RED-CBCT (Verity, Planmed, Helsinki, Finland) was performed in a 210-degree rotation mode. The dose settings for CT imaging were adjusted as close as possible to radiography-equivalent doses (Radiography: Digital imaging plate system PCR Eleva, Philips, Amsterdam, Netherlands). Coming from the standard settings, the setting for kVp was kept and the mA value was adapted so that the resulting dose corresponded to that of radiography. Medium hard kernels were used for image reconstruction, FC30 in MDCT and Hamming in CBCT. Reference standard imaging was performed with MDCT (AquilionOne, Toshiba, Otawara-shi, Japan) in a spiral mode (pitch factor 0.641) based on a high-dose protocol. All images were archived in a Picture Archiving and Communication System (PACS, AGFA Impax 8, Agfa, Mortsel, Belgium). Imaging parameters and radiation doses are shown in Table 1.

**Table 1:** Imaging protocols and radiation doses.

	Settings	Resulting radiation dose	Field of View (FOV)	Matrix in axial images	Slice thickness and sparing in axial images	Pixel size in axial plane
**Radiography**	50 kVp / 2 mAs dorsopalmar 50 kVp / 2.5 mAs lateral	2.5 ± 0.09 mGy				
**MDCT**	100 kVp / 7 mAs	2.31 ± 0.05 mGy	16 x 16 x 12.8 cm	512 x 512	0.2 mm	0.3 mm
**CBCT**	84 kVp / 14.4 mAs	2.17 ± 0.05 mGy	16 x 16 x 13 cm	801 x 801	0.2 mm	0.2 mm
**Reference standard high-dose MDCT**	120 kV / 150 mAs	high dose protocol	16 x 16 x 12.8 cm	512 x 512	0.2 mm	0.3 mm

### Qualitative and quantitative image analysis

Three radiologists with 3, 4 and 5 years of experience independently evaluated the images using a PACS. The raters worked with calibrated displays (RadiForce RX220; EIZO Corp, Hakusan, Ishikawa, Japan). In CT images, window levels were initially set to L/W 350/2000, with the possibility of individual adaption and multi-planar reconstructions. De-identification of the images was performed by deleting any information of modality, protocol and study objects in the Digital Imaging and Communications in Medicine (DICOM) files prior to analysis. As radiography has a typical image appearance, blinding was not possible. Therefore, radiography was analyzed in the first round. Two weeks later, cross-sectional images were handed to the raters in randomized order, containing 5 RED-MDCT and 5 RED-CBCT scans. Another two weeks later, the raters had to analyze the remaining 5 RED-MDCT and 5 RED-CBCT scans. The readers evaluated the images for fractures, joint involvement and were asked to count the number of fragments. Any visible bony part counted as a fragment. There was no cut-off defined for fragment size. A 5-point Likert scale was used to specify the rater´s certainty of their findings (with numbers from 1 to 5 representing 1 = very high certainty up to 5 = very low certainty). The reference standard was performed by two radiologists with 4 and 6 years of experience without knowledge of the index test results. These radiologists performed a consensus reading of the high-dose MDCT examinations with knowledge of the fracturing protocol.

### Statistics

R version 3.1.2 was used for statistical analysis. We determined a p-value < 0.05 to implicate statistical significance. Family-wise error rate was controlled by using the Bonferroni-Holm method [22]. Confidence intervals (CI) were given at a level of 95%. Inter-rater correlation for fractures and joint involvement was analyzed with Krippendorff´s alpha [23]. Bootstrapping of Krippendorff´s alpha was performed with 1000 replicates. Subsequently a one-way ANOVA and post hoc Tukey Honest Significant Differences Test were performed. Sensitivity and specificity for fractures and joint involvement were calculated with Cochrane´s Q and post hoc analysis. Correlation of the number of fragments compared to the reference standard was performed by Pearson and Filon´s z. Comparison of confidence for the rater´s results regarding fracture, joint involvement and number of fragments was calculated with Friedman Test and post hoc Nemenyi Test [24].

## Results

According to the reference standard, 9 out of 140 bones were fractured. In total, there were 5 fractures induced to the phalanges proximales, 2 fractures induced to the phalanges mediales and 2 fractures to the phalanges distales. One fracture had only 2 fragments, another fracture had 3 fragments, 5 fractures had 4 fragments and 2 fractures had 5 fragments. There were 6 fractures with joint involvement.

Inter-rater correlation for fractures was 0.77 for RED-MDCT, 0.73 for RED-CBCT and 0.4 for radiography (p < 0.001). Inter-rater correlation for joint involvement was 0.72 for RED-MDCT, 0.63 for RED-CBCT and 0.39 for radiography (p < 0.001). Inter-rater correlation for fragment count was 0.81 for RED-MDCT, 0.60 for RED-CBCT and 0.36 for radiography (p < 0.001).

For sensitivity of fractures (see Table 2), there was no significant difference between the modalities (p = 0.61). Specificity for fractures (see Table 2) was significantly higher for RED-MDCT (0.96; CI 0.94–0.98) and RED-CBCT (0.96; CI 0.94–0.98) compared to radiography (0.9; CI 0.87–0.93) with p < 0.001.

**Table 2:** Sensitivity and specificity of fractures.

Fracture	Sens	Lower CI	Upper CI	CQ	Spec	Lower CI	Upper CI	CQ	Post hoc comp radiography	Post hoc comp MDCT
**Radiography**	0.71	0.53	0.89	0.61	0.90	0.87	0.93	< 0.001		
**RED-MDCT**	075	0.58	0.92		0.96	0.94	0.98		< 0.001	
**RED-CBCT**	0.71	0.53	0.89		0.96	0.94	0.98		< 0.001	1

For sensitivity of joint involvement (see Table 3), there was no significant difference between the modalities (p = 0.61). Specificity for joint involvement (see Table 3) was significantly higher for RED-MDCT (0.97; CI 0.96–0.99) and RED-CBCT (0.98; CI 0.96–0.99) with p < 0.001.

**Table 3:** Sensitivity and specificity of joint involvement.

Joint Involvement	Sens	Lower CI	Upper CI	CQ	Spec	Lower CI	Upper CI	CQ	Post hoc comp radiography	Post hoc comp MDCT
**Radiography**	0.61	0.39	0.84	0.61	0.93	0.90	0.95	< 0.001		
**RED-MDCT**	0.67	0.45	0.88		0.97	0.96	0.99		0.003	
**RED-CBCT**	0.61	0.39	0.84		0.98	0.96	0.99		0.002	1

Correlation of the number of fragments to the reference standard was significantly higher for RED-MDCT (0.66; CI 0.61–0.71) and RED-CBCT (0.64; CI 0.58–0.7) compared to radiography (0.33; CI 0.24–0.41) with p < 0.001. There was no significant difference between RED-MDCT and RED-CBCT with p = 0.41.

Comparing the rater´s confidence with his findings regarding fractures, RED-MDCT (median = 1) and RED-CBCT (median = 1) performed significantly better than radiography (median = 3) with p < 0.001. Confidence for joint involvement showed the same results.

Comparing the rater´s confidence for the number of fragments, RED-MDCT (median = 1) and RED-CBCT (median = 1) performed better than radiography (median = 3) with p < 0.001.

## Discussion

In this preclinical study on cadaveric hands, we demonstrate that the raters´ confidence in their reporting of finger fractures was higher in RED-MDCT and RED-CBCT compared to radiography. Furthermore, the specificity for fractures and joint involvement was higher in the CT imaging techniques. Sensitivity for fractures and joint involvement, however, did not differ between the modalities.

**Fig 1:**
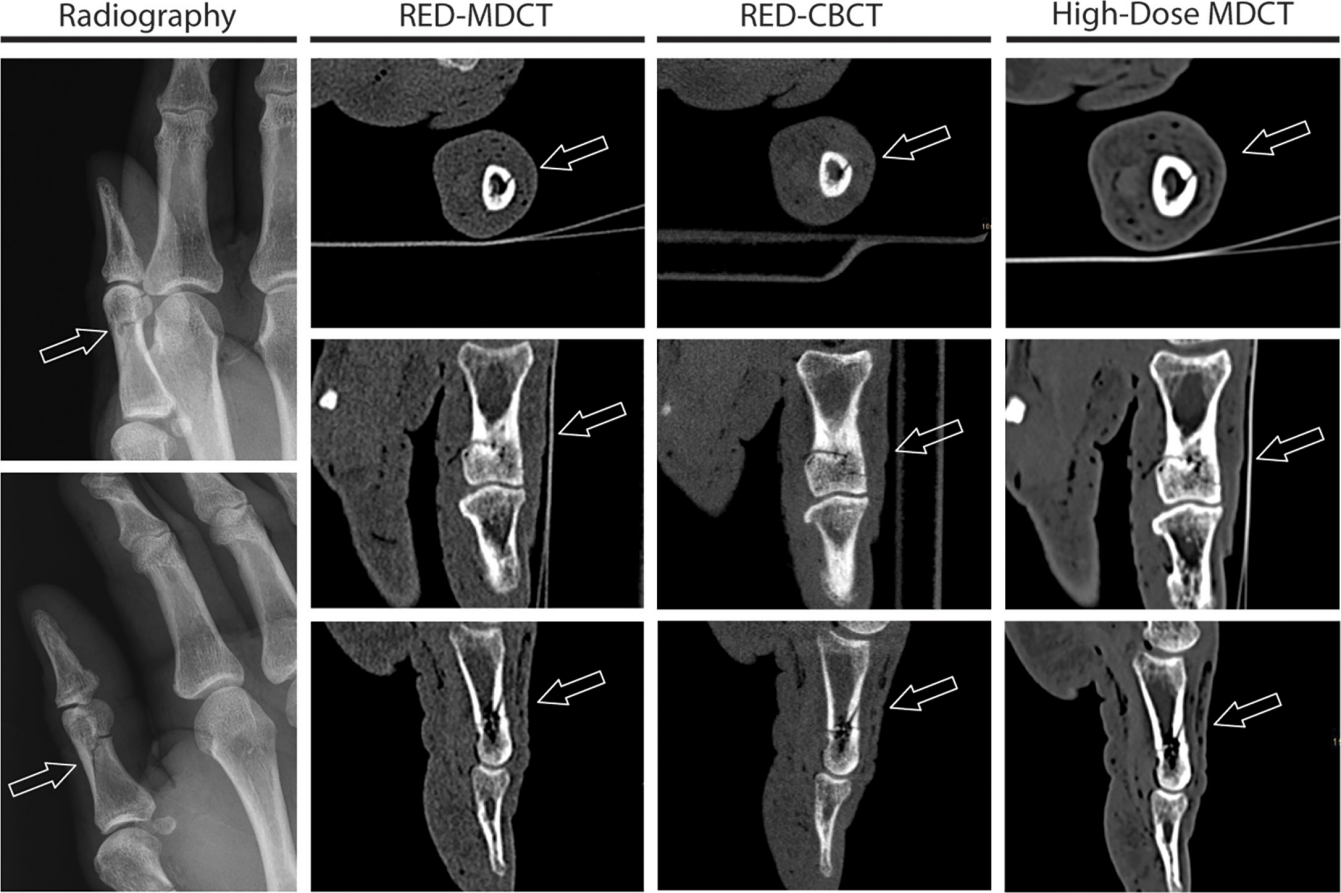
Fracture of Digitus I, Phalanx proximalis (see arrow pointing towards fracture)—Imaging examples with axial, coronal and sagittal reconstructions (top down) for RED-MDCT (second column), RED-CBCT (third column), high-dose MDCT (fourth column) and radiography (first column—p.a., top; and oblique, bottom).

**Fig 2:**
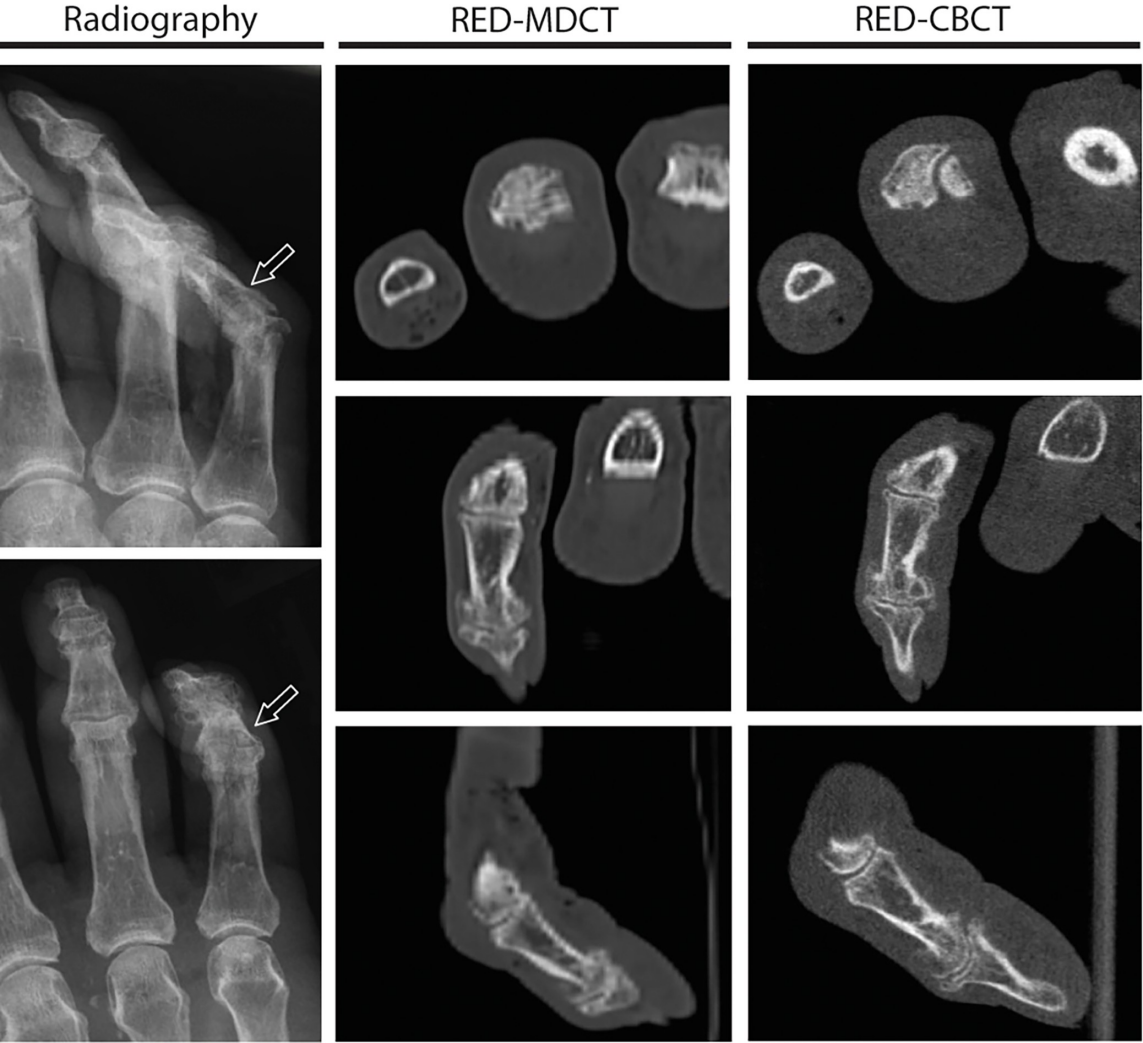
Raters suspected a fracture in radiography in Phalanx media of Digitus V that didn´t exist and therefore didn´t show up in the RED-CT images. Imaging examples with axial, coronal and sagittal reconstructions (top down) for radiography (first column, see arrow pointing towards the suspected fracture), RED-MDCT (second column) and RED-CBCT (third column).

Finger bones are comparably small bony structures. Therefore, the imaging diagnostics of those small bones especially benefit from high spatial resolution. Radiography, however, has a much higher spatial resolution than the sectional imaging methods, which is a clear advantage in this particular region of the body and could explain the similar results regarding the sensitivity for fractures [25]. An essential advantage of the CT imaging is the superimposition-free representation by means of cross-sectional images. However, the superimpositions in finger radiographs are quite limited, which is why this advantage of CT probably does not come to bear when it comes to the detection of finger fractures and their joint involvement (see Figs 1 and 2 for imaging examples).

Between RED-CBCT and RED-MDCT no relevant difference could be found in the parameters of diagnostic accuracy, the modalities are thus to be regarded as equivalent. However, it is noticeable that the sensitivities of the RED-CT for fractures, but also for their joint involvement were relatively low in our study when compared with established values from the literature (Faccioli et al.). It is possible that this may be partly associated with the dose reduction of the RED-CT studies and the associated deterioration of image quality.

On the other hand, the sensitivity of radiography is also relatively low in this study. Possibly, the cause of the relatively low sensitivities is to be found in the structure of our reference standard. Since the fracturing protocol has been incorporated here, fractures that are almost invisible in MDCT might have been included in the reference standard regularly. This in turn might have reduced the sensitivity of the index tests. In addition, it can be assumed that the raters in the study situation with artificial fractures in corpse hands delivered a worse performance than it would have been the case in a real clinical setting. In this respect, the values found in this study for sensitivities are not directly transferable to clinical practice as absolute values. The comparison of the modalities with each other, which was the actual question of the study, is nevertheless possible and valid from our point of view, independent of the absolute values.

The higher certainty of the raters and inter-rater correlation makes it very likely that the diagnostic performance can be improved by RED-CT compared to radiography. Also, the higher specificity for fractures in the RED-CT clearly points in this direction. The better correlation of fragment count with the reference standard and higher specificity for joint involvement in the RED-CT may be indicative of a better ability to correctly classify fractures, which in turn could be relevant to adequate therapy decision. This is particularly important as delayed or wrong diagnosis and treatment could lead to complications such as pseudarthrosis [26] and osteoarthrosis, possibly resulting in higher treatment expenses as well as socio-economic consequences in the long run.

The idea of reducing the radiation dose of CT examinations to the level of radiography examinations has been performed especially in chest imaging, where reduced-dose CT turned out to be a feasible alternative to radiography [10]. A good detection rate of pulmonary nodules and infectious lung disease has been described for these examinations [11,27].

In musculoskeletal imaging, a reduction of the radiation dose has also been described for CT examinations of the wrist mainly by reducing voltage, although the dose values achieved in that study continue to lie above the radiation dose for radiography [12].

However, advantages in the diagnostic performance of CT examinations with equivalent dose to radiography have also been shown for the assessment of carpal fractures [28]. The complex ovoid shape of carpal bones and complex fracture planes make detection of fracture in radiography a challenging task and superimposition-free appearance of cross-sectional images is of clear advantage in the carpus. In the fingers, where anatomic conditions result in less superimposition, interpretation of radiography might be less erroneous. Nevertheless, for finger fractures, an adequate image quality and fracture detection for MDCT and CBCT has been described for regular radiation dose settings [1].

Our data for finger fractures are therefore in line with these studies, in that CT examinations of the fingers are possible with radiography equivalent dose. Also, diagnostics can partly be improved by performing radiography equivalent dose CT examinations of the fingers.

This study, however, has several limitations. Its small number of samples limits it. By chance in rolling the dice, only 9 fractures were induced in 140 bones. The design was chosen to reflect the frequency of finger fractures in everyday clinical practice. However, the low number of fractures led to relatively large confidence intervals regarding the parameters of diagnostic accuracy. Small differences in the measures of sensitivity for fractures might therefore stay undetected. However, it was not possible to increase the number of cadaveric hands for capacity reasons. Also, the fractures induced in this study were of artificial nature and comparability to fractures appearing in clinical practice will be limited, despite the fact that all measures possible in this scenario were taken to accomplish this goal. As we examined cadaveric hands from body donors, who are assumed to be older, our spectrum of hands does not accord with that of e.g. an emergency unit, where also younger persons with finger fractures are treated, keeping in mind that especially younger persons are affected by finger fractures [29].

The aim of this study was to compare dose-equivalent radiography and CT examinations. In order to maintain the condition of dose equivalence, we had to take into account that the same body area was irradiated in all examinations. However, the examination of individual fingers was not possible in radiography due to the stiffness of the cadaveric hands. This is why we always had to examine the entire hand. Hence, the results of our study refer to p.a. and oblique radiographs of the entire hand. Superimposition due to stiffness might have contributed to lower sensitivity for fractures in radiography. Radiography of the finger in two planes is usually performed in the clinical situation of suspected fracture of a single finger. If, however, there is diffuse pain in the hand or several fingers have been injured, p.a. and oblique radiographs of the entire hand are often applied as the imaging method at our institution in order to determine the exact location of an injury and to exclude further fractures. The study thus represents part of the clinical practice at our institution.

Examining cadaveric hands also implicates the appearance of emphysema, although we took every measure to reduce its extent. Emphysema can also develop in fracture regions and therefore could have been an indirect sign for fractures. This, however, is unlikely from our perspective as emphysema also occurred in non-fractured regions. Analysis of motion artifacts was not possible in this experimental setup because cadaveric hands were used. Also, Iterative reconstruction was not available in our CBCT device, a fact that could have led to raising image noise.

In summary, in this phantom study RED-CT demonstrates a partly higher diagnostic accuracy than radiography. Implementing RED-CT in the diagnostic work-up of finger fractures could improve diagnostics, support correct classification and adequate treatment. If a CBCT device is available, it could besides relieve MDCT, whose capacity could then be free for emergency examinations. Clinical studies should be performed to confirm these preliminary results.

## Supporting information

S1 Table PhalTabstop. Table including examinations for every hand by the 3 raters.
(TXT)

S1 File This legend explains the abbreviations and the structure of S1 Table. (DOCX)
