# Supplementary material for: Comparing the diagnostic performance of radiation dose-equivalent radiography, multi-detector computed tomography and cone beam computed tomography for finger fractures – A phantom study
Source: PLoS One. 2019 Mar 5;14(3):e0213339. doi: 10.1371/journal.pone.0213339 (PMC6400385; doi:10.1371/journal.pone.0213339)
Supplement: S1 File — (DOCX) [file pone.0213339.s002.docx]

Legend for our supporting information file

- GROUP: 1, 2 and 3 stand for Phalanx proximalis, media and distalis
- DIG.: Digiti 1-5 for each of the 10 cadaveric hands
- F = fracture
- FS = rater´s confidence in fracture
- G = joint involvement
- GS = rater´s confidence in joint involvement
- D = dislocation
- DS = rater´s confidence in dislocation
- Z = number of fragments
- ZS = rater´s confidence in number of fragments
- there were 3 raters: rater A, B and C (named RA, RB, RC)
- there were 3 modalities: radiography, MDCT and CBCT (named 0, 1, 2)
- each rater had to evaluate the existence of fracture, joint involvement and dislocation: 0 = no, 1 = yes
- each rater had to evaluate the fragment count (1-10)
- each rater had to evaluate the confidence with his findings: 1-5 (with 1 = very high certainty, 5 = very low certainty)

Example:

F_RA_0

Existence of fracture Rater A Modality: radiography

(0 = no, 1 = yes)
